# Flexible males, proactive females: increased boldness/exploration damping with time in male but not female colonists

**DOI:** 10.1098/rsos.240842

**Published:** 2024-12-18

**Authors:** Andrey V. Tchabovsky, Elena N. Surkova, Ludmila E. Savinetskaya, Ivan S. Khropov

**Affiliations:** ^1^ Laboratory for Population Ecology, Severtsov Institute of Ecology and Evolution, Russian Academy of Sciences, 33 Leninskii Pr., Moscow, Russia

**Keywords:** colonization, behavioural trait, phenotypic plasticity, gerbils, landscape change, range expansion

## Abstract

Individuals colonizing new areas during range expansion encounter challenging and unfamiliar environments, suggesting that colonists should differ in behavioural traits from residents of source populations. The colonizer syndrome is supposed to be associated with boldness, exploration, activity and low sociability. We assessed spatial and temporal variation of the colonizer syndrome in an expanding population of midday gerbils (*Meriones meridianus*). Male-first colonists did not differ significantly from residents of the source population, whereas female-first colonists were bolder, faster and more explorative than females from the source population. These findings support a boldness/exploration syndrome as a typical colonizer trait, which appears to be restricted to females in midday gerbils. Males and females also differed in behavioural dynamics after colony establishment. In males, boldness/exploration/sociability peaked in newly founded colonies, then sharply decreased in subsequent generations consistently with decreasing environmental uncertainty in ageing colonies. In females, greater boldness/exploration did not diminish with time post-colonization, i.e. female colonists retained the bold/explorative phenotype in subsequent generations despite facing a less challenging environment. Thus, female colonists, unlike males, carry a specialized behavioural colonizer phenotype corresponding to a proactive behavioural coping strategy. We link sex differences in behavioural traits of colonists to sex-specific life-history strategies.

## 1. Introduction

The propensity to disperse and colonize new areas is believed to be associated with a set of individual traits—genetically and/or developmentally determined—that distinguish dispersers and colonists from residents of the source population [1–11]. If these traits are correlated, then they form a specific dispersal syndrome [12–15]. The propensity to disperse and the ability to transfer to and settle in a new location have been shown to correlate with boldness (risk-taking), exploration (novelty-seeking), activity, low sociability and high aggressiveness [10,16–23]: personality traits associated with the proactive strategy of coping with environmental challenges [24–27]. For example, in sticklebacks (*Gasterosteus aculeatus*) colonizing freshwater environments, dispersing fish are bolder than conspecifics in well-established populations, and boldness decreases with time since establishment [28]. In great tits (*Parus major*), natal dispersal distances correlate positively with exploratory behaviour of individuals and their parents, implying that dispersal and novelty-seeking are linked and heritable [16]. Furthermore, levels of exploratory and risky behaviours in great tits appear to be underpinned by a polymorphism in D4 dopamine receptor gene *Drd4* [29] (but see [30]) associated with novelty-seeking and migration distances in the humans that colonized South America [31]. Dark-eyed juncos (*Junco hyemalis*) colonizing a novel urban environment in southern California are bolder and more explorative than conspecifics from the source population [32]. In western bluebirds *Sialia mexicana*, aggressive males disperse from core populations to the front of invasion in northwestern United States, where they displace local less aggressive mountain bluebirds *Sialia currucoides*, thereby promoting further range expansion [17]. Moreover, the aggression of western bluebird colonists decreased rapidly across consecutive generations owing to selection on heritable low-flexible aggressive phenotypes after colony establishment [18]. The negative relationship between sociability and the propensity to disperse has been shown in females of the yellow-bellied marmot *Marmota flaviventris* [33] and the grey‐sided vole *Myodes rufocanus* [34] as well as in the invasive mosquitofish *Gambusia affinis* [19], thus supporting the social cohesion hypothesis of Bekoff [2]. The existence of consistent specific behavioural traits in colonists indicates that they are not simply a random sample from the source population but are spatially sorted phenotypes with bolder, more explorative or more aggressive individuals found at the edges of expanding ranges, where they face novel and uncertain ecological and social conditions [4,9,17,18,28,35,36].

Nevertheless, the spatial sorting of behavioural phenotypes along the expansion axis is not always revealed or does not necessarily follow a predicted direction. Overall, it remains unclear how widespread the non-random spatial patterns of trait variation are across ranges of populations that have recently expanded [35]. For example, in the gambusia (*G. affinis*), the syndrome of correlated individually consistent traits—supposed to be associated with the propensity and ability to disperse (general activity, exploration and boldness)—is not related to dispersal distance [19]. Among round gobies *Neogobius melanostomus*, fish from recently established populations are less bold than conspecifics from the older population [37]. In invasive bank voles (*Myodes glareolus*) colonizing new areas during the ongoing range expansion in Ireland, individuals at the expansion edge are shyer and more careful explorers compared with conspecifics in the source population, thereby supporting the dangerous-niche hypothesis, predicting that accurate exploring is beneficial in an unfamiliar or unpredictable environment, especially in prey species [11]. Contradictory results obtained on different taxa highlight the importance of species ecology for predicting and explaining spatial patterns of phenotype sorting across expanding ranges [11].

The inconsistencies in the results may also be due to the lack of field studies on short-term dynamics of ongoing colonization processes: very few studies have addressed not only spatial variation of behavioural traits across expanding ranges but also temporal dynamics of behavioural profiles post-colonization (e.g. [11,18,23,38]). With the lack of direct observations at short time intervals, the evidence for behavioural sorting during colonization remains equivocal. Additionally, it is unclear which behavioural types are favourable in different spatial zones of expanding ranges and at different stages of colonization [11,28,38]. Colonization begins with the arrival of first colonists settling in a vacant area, which are known as settlers or pioneers, followed by the flow of joiners: immigrants from the core population [4,14]. Settlers, immigrants and their recruited offspring face different ecological and social challenges because environmental novelty and uncertainty decrease with time after first arrivals with increasing habitat familiarization, social cues and social landscape connectivity [39,40]. Changes in environmental uncertainty after colony establishment imply that colonists’ behaviour may not only differ consistently from that of the residents of the source population (owing to spatial sorting) but also may change with the colony’s age. This change may occur due to adaptation to local ecological and social conditions in colonies in the long term (as in the western bluebird: [18,41]), as well as in the short term because of behavioural flexibility, i.e. a context-dependent behavioural response to novel environmental conditions [11,22]. Behavioural flexibility, as an ability of individuals to adjust their phenotype to a changing environment throughout the lifespan [42], should be beneficial under unpredictable conditions at the front of an expanding range [22]. Often, it is unclear whether differences in behaviour between the edge and core populations reflect different phenotypes or phenotypic plasticity because direct observations of colonization events are rare [17,32]. Direct observations at short time intervals can help disentangle these effects by providing an opportunity to follow short-term changes in colonist behaviour, which otherwise can be overlooked [37,43].

Dispersal-related personality traits such as boldness, exploration, activity and aggressiveness also affect and are affected by sexual selection [35]. Because in mammals, males and females differ in the dispersal propensity, they may be exposed to differential selection pressures during a population expansion, producing sex-specific spatial sorting patterns of behavioural traits [38]. In common voles, males—as the dispersing sex—are more risk averse at the edge of ongoing expansion than males in the source population, whereas females are not spatially sorted [38]. On the other hand, females in mammals are considered a colonizing sex, implying spatial sorting of females with specific life- history and behavioural traits favouring long-distance dispersal and founding of new colonies [44,45]. Studying sex-specific colonizing strategies may contribute to the understanding of the evolution of dispersal and life-history strategies in males and females and mechanisms of species invasions and range expansions. Nonetheless, this intriguing field of colonization research remains underexplored, and we know of only one study on sex-specific colonist traits in mammals [38].

Recent dynamics of rodent populations in the rangelands of Kalmykia (southern Russia) under the influence of human-induced landscape transformations are an example of rapid species range shifts driven by cycles of desertification–steppification [46–52]. In response to the expansion of the tall-grass steppe in the 1990s, the population of the desert-dwelling midday gerbil *Meriones meridianus* first abruptly declined and then collapsed, and by the mid-2010s, its range had contracted to the east [52,53]. The new cycle of desertification launched the expansion of the midday gerbil population in the opposite direction: to the west in 2019, where they began to recolonize re-emerging desert habitats, thereby providing a rare opportunity to observe the colonization process in real time.

In this study, we aimed to assess spatial and temporal variation of behavioural traits of midday gerbils in an expanding population by multi-assay personality tests previously designed to measure boldness, exploration, sociability and docility in gerbils [54]. We tested whether first colonists (pioneers) differ from residents of the source population by being either bold/fast or timid/thorough explorers. A difference would indicate spatial sorting of specific colonist phenotypes or behavioural flexibility driven by environmental effects on pioneers in new areas. Next, we hypothesized that if the behavioural traits of colonists quickly change with the colonies’ age, thus approaching the baseline characteristics of the residents of source populations, then this change indicates a flexible reversible behavioural response to decreasing uncertainty of social and ecological conditions. If, on the contrary, behavioural profiles of colonists remain specific and consistent over generations, then this indicates spatial sorting of specialized colonizer phenotypes. We could not assess the flexibility of behavioural responses as the ability of individuals to adjust their phenotype to a changing environment throughout the lifespan [42] because we tested gerbils within short field sessions in spring, and few adult gerbils survived until the next session. Nevertheless, we assumed that if subsequent generations of gerbils after colony establishment differ from the first pioneers, thus showing patterns similar to residents of the source population, then this difference would probably reflect a flexible reversible response of the first settlers to environmental novelty rather than spatial sorting of the consistent specific colonizer phenotypes. Finally, we determined whether patterns of spatio-temporal variation of behavioural traits differ between males and females. We did not make any specific predictions on the sociability of colonists because previous reports and the theory on the role of sociability in colonization success are the most controversial [10].

## 2. Material and methods

### 2.1. Model species

*Meriones meridianus* is a small (50−60 g of adult body mass), short-lived (life expectancy *ca* eight months), nocturnal–diurnal, mainly granivorous, psammophilous gerbil inhabiting deserts and semi-deserts of Central Asia, Northern China and Southern Russia [55–58]. Midday gerbils live solitarily or in loose aggregations: males and females are not territorial, do not form pair bonds, are socially promiscuous, interact rarely, display little agonistic or amicable behaviour and on the whole are socially indifferent [59–62]. Their breeding season in Kalmykia typically lasts from early April to mid-September. From mid-April to mid-May, when the tests were performed, the population consisted mainly of adults that were born in a previous year and overwintered and some young individuals (excluded from the analysis due to small sample sizes).

### 2.2. Study area

It is located in semi-desert rangelands of southeastern Kalmykia and neighbouring east Astrakhan Oblast (southern Russia). Kalmykia represents the very northwestern edge of this species’ range, where population dynamics of gerbils are driven by cycles of desertification–steppification [47,52,53,57]. Here, gerbils burrow in patchily distributed desert habitats on semi-stabilized hilly sands isolated by a matrix of unsuitable semi-desert loamy plains or tall-grassed stabilized sandy patches. We subdivided the entire area into two zones: a western zone (at the edge of this species’ range) and an eastern zone (extending towards the range core). The western zone spans 50 km from the range border in the west to the east and features more or less isolated sandy patches suitable for gerbils; these patches have been monitored annually twice a year since 1994 (in mid-April to mid-May and mid-September to mid-October). In 2017, the local population collapsed, and gerbils subsequently disappeared from the entire western zone. We continued monitoring the abandoned western zone twice a year, by inspecting sandy patches potentially suitable for gerbils for the presence of burrows to be prepared for the gerbils to come back following the new cycle of desertification [52]. The eastern zone, where gerbils have persisted since the 1960s [52,63], extends further to the east and northeast, towards the range core, for another 100 km and has been monitored since 2017. The invasion of gerbils into the western zone began in the autumn of 2019, with the first few colonists appearing at two sites at its eastern borders. Since then, new colonies have emerged in the western zone every year, as discovered by regular inspections of all monitored locations during spring and autumn sessions [52].

### 2.3. Trapping procedure

In this study, we used data collected in 2022−2023 in spring (mid-April to mid-May). We trapped gerbils with modified Shchipanov’s non-commercial wired live traps [64] baited with sunflower seeds and placed at entrances of active burrows at the beginning of evening activity (approx. 19.00). We set traps for 3−5 h and checked them once an hour. Captured gerbils were carried in traps to a mobile field laboratory located 50−200 m away, where tests and other manipulations were performed. We marked each gerbil with an animal ID microchip compliant transponder (1.25 × 7 mm; FOFIA, Wuxi, China).

### 2.4. Experimental procedure

The entire procedure—designed to assess personality traits in gerbils [54]—included four consecutive tests (table 1). The first test evaluated a single metric: the activity of a gerbil in a handling bag. The second, third and fourth tests were composite, included multiple measurements and were carried out in suites of consecutive assays within the same experimental arena.

**Table 1.** The sequence of tests, recorded behaviours, associated traits and metrics.

test	behaviours	behavioural traits	metrics
(A) bag	immobility	docility	(1) time spent immobile (s)
(B) dark–light/startle/novel object (DL/S/NO)			latencies (s):
DL/S	emergence from a shelter	boldness	(2) body out (BO)
NO	sniffing an object	exploration	(3) contact object (CO)
(C) dark–light/elevated platform (DL/EP)			latencies (s):
DL	emergence from the shelter	boldness	(4) body out (BO)
EP	climbing down	anxiety	(5) climb down (CD)
(D) dark–light/stranger (DL/STR)			latencies (s):
DL	emergence from the shelter	boldness	(6) body out (BO)
STR	sniffing a stranger	sociability	(7) contact stranger (CS)
B, C and D combined			latency (s):
		trial execution speed	(8) execution time (ET)

Three composite tests (B–D) included one universal behavioural measure (BO) and one test-specific measure: latency to the final event (FE). ET: the sum of latencies to FEs in three composite tests. Note that boldness, exploration, sociability and trial execution speed were measured by means of metrics opposite to the measured traits—latencies to events (BO, CO, CS and their sum, respectively)—and therefore, the shorter the latencies, the bolder, more explorative, more sociable and faster the individuals are. Docility and anxiety were evaluated with direct metrics: the more time spent immobile and the longer the latency to CD, the more docile and anxious the individual is, respectively.

First, to assess docility (a response to restraint), we placed a small cotton handling bag (35 × 25 cm) with a gerbil inside in a plastic bowl and measured the time (in s) the gerbil spent immobile during 1 min. Then, the animal was released into an opaque metal box (25 × 7 × 7 cm) and placed in an illuminated plastic arena (78 × 56 × 43 cm) with a floor covered with sand. After 1 min of acclimatization, we opened the door of the box remotely to start the dark–light startle/novel object test. When the gerbil showed its head from the box, we noisily dropped a metal cylinder (5 × 4 cm) on the opposite side of the arena and recorded latency from the start of the experiment until the gerbil emerged from the box with all four limbs (‘body out’: BO) as a measure of boldness and then, the time it took the gerbil to approach and sniff the dropped cylinder (‘contact with novel object’: CO) as a measure of exploration.

At the next stage, we returned the gerbil to the box and placed it on an elevated (16 cm) Plexiglas transparent platform (50 × 8 cm) with no walls within the same illuminated arena. After 1 min of habituation, we opened the door and recorded latency to a BO event, and then the time it took the gerbil to climb down (CD), as a measure of anxiety (fear-related behaviour). At the final stage, we removed the platform, returned the gerbil to the box and placed a wire-mesh cage (15 × 6.5 × 6.5 cm) with an unfamiliar adult male on the arena’s side opposite the box with a tested gerbil. After 1 min of habituation, we opened the door and measured latency to a BO event and then the time it took the gerbil to approach and contact (sniff) the stranger through the wire mesh (‘contact stranger’: CS) to assess sociability. We used three adult males as strangers: one in 2022 and two in 2023, when two teams worked in parallel at the same sites. Male strangers were trapped in a distant location and had—normal for adult males in spring—body weight of 55−60 g, scrotal testes and active ventral glands. The effect of stranger ID on latencies to BO and CS in linear mixed-effect models (LMMs) (with tested animal ID fitted as a random term) was insignificant (*p* > 0.5). During the entire procedure in the arena, we did not handle gerbils. To catch a gerbil and place it back in the experimental box between stages of the experiment, we moved a trap with an open entrance close to the animal. In most cases, the gerbil readily entered the trap, and then we released it back into the experimental box.

If an animal did not move its full body outside the box or did not climb down or did not approach the object or stranger during 5 min of the test, then the latencies were set to 300 s. At the end of the trial, the set-up and equipment were cleaned with 70% ethanol, and the floor of the arena was covered with fresh sand before introducing the next animal. In total, we performed 180 test trials (87 on 71 males and 93 on 73 females). More details of the experimental procedure can be found elsewhere [54].

### 2.5. Data analysis

#### 2.5.1. Classification of colonies

We registered a colonizing event if gerbils settled (in spring or the following autumn) in a patch that had remained vacant (as indicated by the lack of burrows) since 2017. Adult gerbils in newly founded colonies represented first settlers (pioneers). The colonies of ages 1, 2 and 3 years, i.e. the same colonies in subsequent years after the founding event, typically contained no settlers and consisted of increasing numbers of locally born gerbils and probably new immigrants (joiners). These three categories of gerbils experience various levels of environmental uncertainty. Settlers face an unfamiliar environment void of conspecifics, burrows or other elements of a social landscape. Joiners immigrate to the habitats that are unfamiliar to them but are already colonized by other gerbils with established burrow networks and social cues. For locally born gerbils, colonies are their native and familiar sites. Over time, after colonies have been established, the social landscape and burrow network should develop, making the environment more predictable and safer [65]. Therefore, new colonies differed intrinsically from the source population and from colonies of age 1+ in composition (and consisted of first settlers) and featured higher environmental uncertainty and novelty. Thus, to assess spatio-temporal variation of behavioural traits during colonization, we compared behavioural measures between gerbils residing in the source population, new colonies and the colonies of ages 1 and 2−3 years. We pooled data for colonies of ages 2 and 3 years because the sample of colonies of age 3 years was small. Sample sizes and numbers of sites with tested gerbils for each residence category are shown in table 2; the list of sites with coordinates is provided in electronic supplementary material, table S1.

**Table 2.** Sample sizes for the residence categories of gerbils.

residence category	no. of test trials and no. of tested individuals (in parentheses)		no. of locations	average distance between locations (km)
	males	females		
source (s)	13 (10)	14 (12)	5	67.9
new colonies (nc)	12 (10)	17 (12)	4	1.9
1 year old colonies (c1)	20 (17)	27 (22)	10	10.9
2−3 year old colonies (c2−3)	19 (17)	12 (12)	6	12.7
total	64 (54)	70 (56)^a^	25	

^a^ Two females were tested in two subsequent years: one in samples nc and c1 and the other one in samples c1 and c2−3.

#### 2.5.2. Statistical analysis

First, we tested for differences in eight primary behavioural metrics (table 1) between residents of the source population and first colonists, i.e. gerbils from the newly established colonies. If an animal did not peek out of the box at all (did not show its head) during any stage of the trial in the arena, then we excluded this stage from the analysis of respective variables. In addition, we excluded data from a ‘climb down’ event if a gerbil simply fell off the platform. Thus, sample sizes could vary between primary variables. Regarding trial execution time, we excluded one gerbil that did not peek out (did not show its head) at all four stages, but we included others who peeked out at least at one stage. Primary metrics assess boldness/exploration in different contexts and may or may not be correlated. Then, to analyse temporal dynamics of behavioural traits during colonization, we used composite variables obtained with principal component analysis (PCA) from the set of primary behavioural metrics; this approach is a common and valid way to reduce the dimensionality of multi-collinear datasets in behavioural research [66] and to combine correlated behaviours into independent behavioural axes (syndromes), i.e. traits with different motivational backgrounds [67].

PCA was conducted using R package *psych* [68] separately for males and females having complete datasets with no missing values of any of the primary variables (table 1; trial execution time was not included in PCA because this metric is a combination of three variables: latencies to final events). Among 110 individuals (54 males and 56 females) included in the dataset, 24 (10 and 14) were tested twice, and only two of them (both were females) in different (consecutive) years. The intertest interval for the other 22 individuals was on average 4.7 days. We included repeated measurements from the same individual in the dataset for PCA: a common approach in behavioural and personality research [67], which, however, does not take into account statistical non-independence of repeated-measures datasets [66]. The issue of non-independent data for PCA in behavioural science has no clear solution, and alternative approaches (e.g. confirmatory factor analysis) may require large sample sizes [69]. Thus, including individuals with a single behavioural measurement and individuals with two or more repeated measurements in the dataset is a common practice, which, in addition, enhances statistical power (e.g. [67,70]). We believe that the repeated measurements could not strongly influence principal component (PC) structure owing to pseudoreplicates because only a few individuals were tested repeatedly and twice at maximum (table 2). To control for pseudoreplicates, in further analyses, we included animal identity in models as a random factor.

PCs were interpreted as composite behavioural responses according to the loadings of primary variables (greater than 0.6 in absolute value as recommended for small sample sizes: [66]). Each animal was assigned scores from each of the retained PCs. PC scores served as response variables to compare behavioural profiles between gerbils from the source population, new colonies and the colonies of ages 1 and 2−3.

Statistical analysis was performed in R v. 4.2.3 [71]. We assessed the variation of primary behavioural metrics as well as composite variables derived via PCA between the source population and colonies in adult males and females by constructing separate LMMs with a restricted maximum-likelihood (REML) method implemented in the *lmer* function of R package *lme4* [72]. Primary behavioural metrics and PCs were fitted to the models as response variables, while the residence category of gerbils (gerbils from the source population, new colonies or colonies of ages 1 and 2−3 years) was included as a predictor, and animal identity as a random factor. We mainly aimed to assess differences in behaviour between emerging colonies and the source population as well as changes in behaviour with colony age after foundation. Thus, we considered the new colonies a reference group in LMMs. For post hoc pairwise comparisons between colonies of different ages and the source population, we used the *emmeans* R package [73] with Tukey-adjusted *p* values for multiple comparisons. Prior to analyses, all measured data were ln(*x*+1)-transformed to improve normality.

## 3. Results

### 3.1. New colonies versus the source population

**Figure 1.**
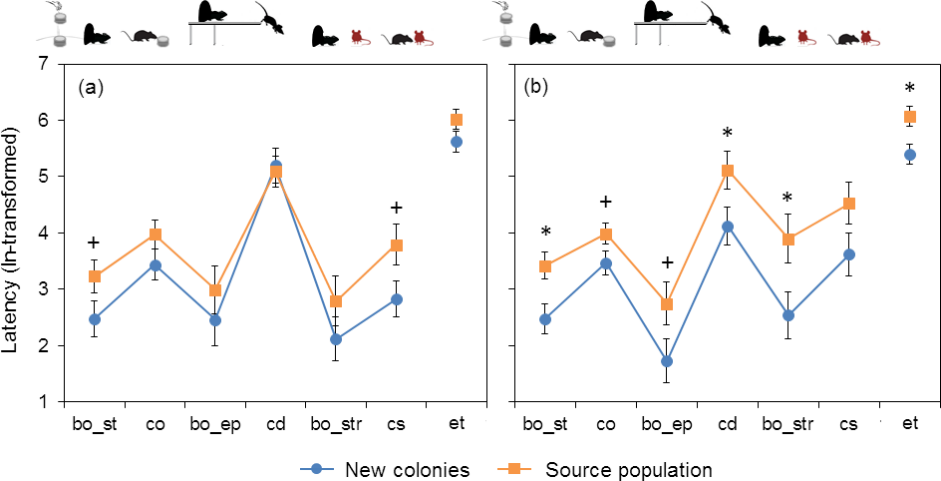
Mean ± s.e. of latencies (ln-transformed) to consecutive events in the composite tests (see table 1) for males (*a*) and females (*b*) from new colonies and the source population. Asterisks and plus signs indicate significant (*p* < 0.05) and marginally significant differences (*p* < 0.1), respectively, as revealed by LMMs (see table 3). bo: body out, co: contact with object, cd: climb down, cs: contact with stranger, et: trial execution time, st: startle test, ep: elevated platform test, str: stranger test.

Time spent immobile in the bag test did not differ between the new colonies and the source population, either in males or females (table 3). Male colonists and residents of the source population showed similar latencies to events at most stages of the composite test. Nonetheless, in emerging from the box during the startle test and in contacting a stranger, they tended to be faster than residents of the source population (figure 1, table 3). Females from new colonies were faster at all stages of the composite test compared with their counterparts from the source population, and unlike males, exhibited significant differences in many metrics. They were bolder as indicated by significantly shorter latencies to show the full body at the dark–light stages of the startle test and the stranger test as well as in the elevated platform test (the difference was close to significance). They also tended to be faster in exploring the novel object and were significantly faster in climbing down from the elevated platform and in executing the entire trial (table 3, figure 1). Random effects of animal ID were insignificant in all models except for immobility in males (likelihood ratio test: *χ*^2^ = 16.5, *p* < 0.0001).

**Table 3.** Coefficients of the LMMs (*B*  ±  s.e.) assessing the effect of the residence category of gerbils (the source population or new colonies) on behavioural metrics in adult males and adult females. Numbers of test trials and of tested individuals are shown in parentheses as trials/individuals. IMMOB: time spent immobile in the bag, BO: body out, CO: contact with object, CD: climb down, CS: contact with stranger, ET: trial execution time, ST: startle test, EP: elevated platform test, STR: stranger test.

fixed effects	adult males			adult females		
behaviour	*B* ± s.e.	*t*	*p*	*B* ± s.e.	*t*	*p*
IMMOB (s)	0.03 ± 0.09 (39/31)	0.3	0.7	−0.04 ± 0.12 (39/32)	−0.3	0.8
BO_ST (s)	*0.76 ± 0.43 (37/30)*	*1.8*	*0.09*	**0.94 ± 0.36** (**39/32**)	**2.6**	**0.01**
CO (s)	0.55 ± 0.38 (37/30)	1.4	0.2	*0.51 ± 0.27 (39/32)*	*1.9*	*0.07*
BO_EP (s)	0.54 ± 0.63 (35/28)	0.9	0.4	*1.02 ± 0.55 (37/30)*	*1.9*	*0.07*
CD (s)	−0.11 ± 0.41 (31/25)	−0.3	0.8	**0.99 ± 0.47** (**36/29**)	**2.1**	**0.045**
BO_STR (s)	0.67 ± 0.58 (29/24)	1.1	0.3	**1.36 ± 0.59** (**33/26**)	**2.3**	**0.03**
CS (s)	*0.95 ± 0.48 (29/24)*	*2.0*	*0.058*	0.91 ± 0.52 (33/26)	1.7	0.1
ET (s)	0.40 ± 0.26 (38/31)	1.5	0.1	**0.67 ± 0.25** (**39/32**)	**2.7**	**0.01**

Note that boldness, exploration, sociability and trial execution speed were measured by means of metrics opposite to the measured traits—latencies to events (BO, CO, CS and their sum)—and therefore the shorter the latencies, the bolder, more explorative, more sociable and faster the individuals are. Docility and anxiety were measured with the direct metrics: the more time spent immobile and the longer the latency to CD, the more docile and anxious the individual is, respectively. New colonies were the reference group, s: source population. Significant (*p* < 0.05) and marginally significant effects (*p* < 0.1) are highlighted in bold and italic, respectively. Animal ID was added as a random term to all models.

#### 3.1.1. Temporal dynamics of behavioural traits post-colonization

PCA of primary behavioural metrics when conducted separately for males and females produced three PCs with eigenvalues greater than or equal to 1.0 for each sex, which in total explained 71% and 74% of the variance, respectively (table 4). In males, PC1 combined short latencies to BO in the startle test and stranger test and to contact with the novel object or the stranger; this PC was labelled ‘boldness/exploration/sociability’. PC2 was mainly associated with short latency to climbing down from the platform, indicating low anxiety, and was termed ‘confidence’. PC3 was mainly related to low activity in the bag, i.e. reflected docility. In females, PC1 combined short latencies to show the full body in all three types of dark–light emergence assay and short latency to contact with the object; this PC was named ‘boldness/exploration’. PC2 was mainly associated with short latency to contact with a stranger and to show the full body in the same test, i.e. reflected sociability, whereas PC3 correlated with short latency to climb down, i.e. indicated low anxiety and was termed ‘confidence’.

**Table 4.** Results of PCA (PCA loadings) of primary behavioural metrics. IMMOB: time spent immobile in the bag, BO: body out, CO: contact with the object, CD: climb down, CS: contact with a stranger. ST: startle test, EP: elevated platform test, STR: stranger test.

	adult males			adult females		
metrics	PC1	PC2	PC3	PC1	PC2	PC3
IMMOB	0.02	−0.45	**0.79**	−0.44	0.18	0.50
BO_ST	**−0.79**	0.40	0.24	**−0.81**	0.28	0.13
CO	*−0.59*	0.54	0.16	**−0.81**	0.27	0.22
BO_EP	−0.25	−0.51	−0.50	**−0.64**	0.43	−0.27
CD	−0.17	**−0.69**	0.20	−0.45	0.07	**−0.77**
BO_STR	**−0.84**	−0.16	−0.06	**−0.71**	*−0.59*	−0.02
CS	**−0.83**	−0.32	−0.15	*−0.58*	**−0.72**	0.04
eigenvalue	2.5	1.5	1.0	3.0	1.3	1.0
% of total variance	35.0	21.7	14.7	42.3	17.9	14.0
PC label	boldness/ exploration/sociability	confidence	docility	boldness/ exploration	sociability	confidence

PCA loadings greater than 0.6 and close to 0.6 in absolute value are boldfaced or italic, respectively. Pairwise correlations between primary variables did not exceed 0.8, and all of them were included in the analysis. Note that boldness, exploration and sociability were measured using metrics opposite to the measured traits—the latencies to events (BO, CO and CS, respectively)—and therefore, the shorter the latencies, the bolder, more explorative and more sociable the individual is. Docility and anxiety were assessed with direct metrics: the more time spent immobile and the longer the latency to CD, the more docile and anxious the individual is, respectively. Therefore, negative (positive) loadings of the relevant primary variables (BO, CO and CS) indicate positive (negative) correlations with, respectively, boldness, exploration and sociability. The sign of loadings for immobility and CD denotes the direction of correlation with docility and anxiety, respectively.

**Figure 2.**
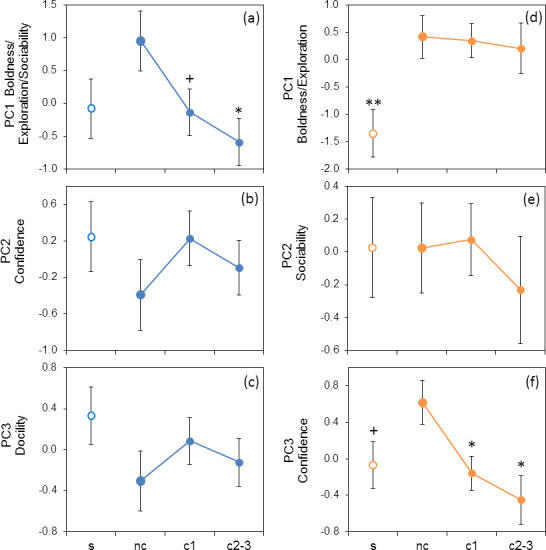
Variation of behavioural traits between the source population (s, open markers), newly founded colonies (nc) and colonies of age 1 year (c1) and 2−3 years (c2−3) for adult males (*a–c*) and adult females (*d–f*). Means ± s.e. of PCs scores (see table 4). Enlarged circles indicate the reference group (nc) in an LMM. Asterisks and plus signs respectively show significant (*p* < 0.05) and nearly significant (*p* < 0.1) differences from the reference group.

In males, PC1 scores (boldness/exploration/sociability) varied with colony age, showed a marginally significant decrease at 1 year after colony foundation and were significantly lower in colonies of age 2−3 years than in new colonies, where they were the highest (table 5, figure 2). Although PC1 scores were also higher in new colonies as compared with the source population, the difference was insignificant. Pairwise comparisons did not reveal differences between 1-year-old or 2- to 3-year-old colonies and the source population (*p* > 0.8). PC2 (confidence) and PC3 (docility) did not differ between the new colonies and source population or between new and older colonies. In females, PC1 scores (boldness/exploration) were significantly higher in new colonies than in the source population and did not significantly change with colony age, thereby remaining higher in 1-year-old colonies (the difference is significant: *p* = 0.01) and 2- to 3-year-old colonies (marginal significance: *p* = 0.08) than in the source population as evidenced by the post hoc comparisons. PC2 (sociability) did not vary across residence categories of females, whereas PC3 (confidence) was significantly higher in new colonies than in older colonies and marginally significantly higher than in the source population but did not differ between the source population and 1-year-old or 2- to 3-year-old colonies (post hoc test: *p* > 0.7). Random effects of animal ID were insignificant in all models.

**Table 5.** Coefficients of the LMMs (*B*  ±  s.e.) assessing the effect of the residence category of gerbils (the source population, new colonies, colonies of age 1 and colonies of age 2−3 years) on the composite behavioural traits derived from PCA (see table 4) in adult males and adult females. Numbers of test trials and of tested individuals are shown in parentheses as trials/individuals.

	adult males (64/54)			adult females (70/56)		
fixed effects	*B* ± s.e.	*t*	*p*	*B* ± s.e.	*t*	*p*
	PC1: boldness/exploration/sociability			PC1: boldness/exploration		
c1	*−1.09 ± 0.58*	*−1.86*	*0.069*	−0.07 ± 0.50	−0.15	0.9
c2−3	**−1.54 ± 0.59**	**−2.63**	**0.01**	−0.21 ± 0.61	−0.35	0.7
s	−1.03 ± 0.65	−1.59	0.1	**−1.77 ± 0.58**	**−3.04**	**0.003**
	PC2: confidence			PC2: sociability		
c1	0.62 ± 0.49	1.26	0.2	0.05 ± 0.35	0.15	0.9
c2−3	0.30 ± 0.49	0.60	0.5	−0.26 ± 0.43	−0.60	0.6
s	0.64 ± 0.55	1.17	0.3	0.00 ± 0.41	0.00	1.0
	PC3: docility			PC3: confidence		
c1	0.39 ± 0.37	1.06	0.3	**−0.78 ± 0.30**	**−2.56**	**0.01**
c2−3	0.18 ± 0.37	0.49	0.6	**−1.07 ± 0.36**	**−2.96**	**0.004**
s	0.64 ± 0.41	1.57	0.1	*−0.68 ± 0.35*	*−1.93*	*0.058*

New colonies were the reference group. c1: colonies of age 1 year, c2−3, colonies of age 2−3 years, s: source population. Animal ID was added as a random term to all models. Significant (*p* < 0.05) and marginally significant (*p* < 0.1) effects are boldfaced or italic, respectively.

## 4. Discussion

In this study on first stages of colonization of vacant habitats by midday gerbils during range expansion, we found sex-specific patterns of (i) behavioural differences between residents of the source population and first colonists as well as (ii) short-term temporal dynamics of the behavioural traits post-colonization. To our knowledge, this is the first study on short-term dynamics of behavioural patterns of colonists in mammals during first stages of colonization.

### 4.1. Spatio-temporal dynamics of behavioural traits in an expanding population

Male-first colonists did not significantly differ in primary metrics or composite behavioural traits from residents of the source population (tables 3 and 5, figures 1 and 2). Nevertheless, they tended to be faster in the startle test and in contacting a stranger, i.e. they exhibited somewhat higher boldness and lower social shyness. In contrast to males, female-first colonists differed consistently from residents of the source population in being bolder and more confident in different contexts (both non-social and social), as indicated by shorter latencies to emerge in the three types of dark–light assay and latency to climb down from the platform. They were also faster in inspecting a novel object and executing the entire trial (table 3, figure 1). These differences in primary metrics are supported by the analysis of composite variables: female-first colonists received higher scores on boldness/exploration and confidence than did residents of the source population (table 5, figure 2). These results support the boldness/exploration syndrome as a specific trait of dispersers and colonists, universal for many—though not all—species [9,10,14,16,20–22,28,36]. In midday gerbils in our work, however, the boldness/exploration syndrome appeared to be a female-specific characteristic of the colonizing population.

A different pattern was found in another study on colonizing rodents, the bank voles in Ireland [38]. Female vole colonists did not show behavioural variation across an expanding range, whereas male colonists were shyer and slower explorers than residents of the core population. In that paper, however, new colonies of ages 1−4 years were merged in their sample; therefore, short-term changes in behaviour after colony establishment could be missed. If we had merged newly established and older colonies, then we would have overlooked post-colonization behavioural changes in males: a tendency for an increase and then an abrupt drop of boldness/exploration 1 year after colony establishment.

The absence of differences in sociability between females from the source population and from colonies, combined with only a weak tendency for lower social shyness in male colonists, adds to the ambiguity of results on the role of individual sociability in colonization success [10,43], which may vary among species due to species specificity of social behaviour. Previously, we have demonstrated that sociability is a consistent personality trait in *M. meridianus* [54,62] and according to the present study seems unrelated to colonization process. We attribute this finding to overall low sociability as a species-specific trait of this socially indifferent gerbil species [59–61]. Midday gerbils have no preferences between familiar and unfamiliar conspecifics in preference tests [62], suggesting that their response to social novelty in colonies is weak.

Males and females exhibited sex-specific patterns not only in the differences between the source population and first colonists (strong differences in females and weak in males) but also in temporal dynamics of behavioural traits after colony establishment (figure 2). In males, boldness/exploration/sociability peaked in newly founded colonies, then sharply declined in subsequent generations, reaching the baseline of the source population. In other words, males in colonies 1–3 years after establishment became as timid as residents of the source population. This dynamic pattern follows changes in environmental challenges and uncertainty, which should be the highest for the first settlers and diminish with the colonies’ age with increasing habitat familiarization, social cues and social landscape connectivity as well as with the development of the burrow network [39,40,65]. The dynamic behavioural response of the male population of colonists to changes in environmental uncertainty suggests that they do not carry specific and consistent colonizer phenotypes persisting across generations. Instead, behavioural changes in the male population of colonists over time may reflect adaptive phenotypic plasticity based on context-dependent variation of motivation to take risks and explore [4,22,65,74], helping male pioneers explore, cope with and settle in unfamiliar ecological and social environments and adjust behavioural responses of next generations of male colonists to the decreasing environmental uncertainty.

Given that in midday gerbils, dispersal—just as in most mammal species—is male-biased, we cannot rule out the possibility that the alterations of male behaviour after colony establishment do not reflect behavioural flexibility but result from the influx of immigrants (joiners) [9,14] carrying more timid phenotypes as compared with the bolder pioneers. Nevertheless, we believe that the reinforcement of colonies by immigrants could not considerably contribute to behavioural changes in the male population of colonists. First, midday gerbils—males and females—manifest strong site fidelity and low migration rates, as shown in removal experiments [61]. Few gerbils from intact habitats invaded adjacent depopulated habitats in the work just cited, and the population recovery went on very slowly, mainly due to recruitment of the locally born young. Then, long-distance movements involved in colonization may be more balanced between sexes or even biased towards females [45,75]. Large influx of males should have biased the sex ratio in colonies; however, an adult sex ratio in the colonizing population did not vary over time and was only slightly more male-biased than that in the source population (females/males: 1.10, 1.05, 1.10 and 1.3 for the new colonies, 1-year-old, 2- to 3-year-old colonies and the source population, respectively [unpublished data]). Finally, the female fertility rate and the recruitment of young in the colonies were high and significantly higher than those in the source population [52]. Thus, male immigrants were unlikely to contribute much to the population of the ageing colonies, which are therefore mostly composed of locally born and recruited gerbils (i.e. descendants of the pioneers).

In female colonists, however, greater boldness/exploration did not diminish with time post-colonization despite declining environmental uncertainty and the growing number of locally born and recruited females (figure 2*d*). In other words, females have retained the bold/explorative phenotype of the first settlers, distinct from timid female residents of the core range, in subsequent generations despite a less challenging and less uncertain environment. This finding implies that female colonists, unlike males, carry a rigid specialized behavioural colonizer phenotype corresponding to a proactive coping strategy [24–27].

In contrast to the boldness/exploration syndrome, confidence (as measured by the elevated platform test), which represents an independent behavioural axis in both sexes as shown by PCA, decreased with time in females after peaking in new colonies (figure 2*f*). Thus, confident behaviour on the elevated platform appears to be a distinct form of risk-taking behaviour in colonists that is independent of boldness measured in other contexts. We do not have a clear explanation for why later generations of female colonists became more cautious in climbing down. We can only theorize that the low confidence on the platform in older generations of female colonists reflects weaker motivation to move as the environment becomes more familiar. Whatever the reason, the female population of colonists exhibited plasticity of this behaviour post-colonization.

We detected no differences in docility between the first colonists and residents of the source population or colonists of later generations in either males or females. This finding contradicts the hypothesis that population expansion selects for docile (non-aggressive) phenotypes, which are more likely to colonize vacant habitats [76]. Previously, we have reported that docility (as a response to restraint) is not correlated with boldness in *M. meridianus* [54], consistent with other articles showing its independence from other personality traits [77]. Probably, the docility measured as a specific response to restraint represents a distinct behavioural trait irrelevant to colonization success.

### 4.2. Plastic versus consistent colonist phenotypes of males and females

Dispersal and colonization processes include the same three stages (emigration, transfer and settlement in a new location) but are distinct in required specific individual traits and costs as well as in ecological and evolutionary consequences [44,78]. Unlike dispersal within a population’s geographic range, colonization is typically associated with long-distance movements beyond the genetic neighbourhood and/or population geographic range [79,80] and often implies crossing a matrix of fragmented landscapes to settle in suitable but unfamiliar habitats free from conspecifics [81]: gaps in a social landscape. Consistently with the dangerous-habitat hypothesis, pioneers should be shy and slow explorers, and in mammals, risk aversion should be higher in males as the dispersing sex and under strong selection during colonization of unfamiliar areas void of conspecifics, as shown in bank voles of Ireland [11,38].

On the contrary, in midday gerbils, we found that males did not behaviourally differ between the core and edge populations (though pioneers tended to be bolder and faster), becoming timider with time after colony establishment. This observation contradicts the dangerous-niche hypothesis on spatial sorting of risk-averse males in an expanding population. Moreover, females did exhibit spatial sorting with less risk-averse more explorative proactive phenotypes that were found in the edge population and were retained in subsequent female generations of colonists. Eccard *et al*.’s idea [38] that non-dispersing sex should not be selected for risk aversion does not explain the spatial sorting of typically philopatric females of midday gerbils, i.e. why female colonists are consistently bolder and more explorative than residents of the source population.

Just as Eccard *et al*., we relate sex-specific patterns of plasticity and spatio-temporal dynamics of behavioural traits in an expanding population of gerbils to sex-specific life-history strategies and space use but from another perspective. Sexual conflict theory posits that contrary to the ‘risk aversion hypothesis’, males unlike females are selected for enhanced competitive abilities and risk-taking behaviour, which increases their reproductive success [82,83]. Indeed, personality research shows that males are typically more explorative, bolder and less risk averse and experience higher predation risk than females do ([32,84], but see [85]). In mammals, males are the dispersing sex, while dispersal is risky [86]. Taken together, these observations imply that males are likely to face more variable and less predictable environments, which may favour the flexibility of behaviour (i.e. responding adequately to immediate challenges and changing conditions).

In mammals, colonizing females may differ in behaviour from resident philopatric females or females dispersing within the population because colonization requires specific traits and costs [44]. Colonizers are expected to be risk prone and suffer lower survival, especially in the fragmented landscape, as shown in the individual-based models [79,87]. Long-distance dispersal beyond a genetic neighbourhood to occupy vacant habitats should be a difficult and costly challenge for typically philopatric and kin-oriented mammalian females [88–90]. For strongly philopatric site-attached females of the midday gerbil [61], crossing the matrix of the fragmented landscape of Kalmykian rangelands should be a major challenge. Therefore, the bold explorative colonizer phenotype, stable over subsequent generations of colonist females and uncoupled from the diminishing environmental uncertainty, may be a female-specific individual trait favouring colonization.

Two sex-specific behavioural phenotypes of colonists—a flexible trait in males and a rigid proactive colonizer syndrome in females—match two alternative models explaining colonization and invasion success: behavioural plasticity and behavioural syndrome [4,9,14,38,74,91]. The former helps respond quickly to changing and unpredictable environmental conditions. The latter suggests that colonists are not simply a random sample from the source population but are behaviourally pre-adapted to facing the stressful and uncertain risky environments in colonized novel areas and thus are spatially sorted. One essential inference from our study is that these two contrasting strategies may be sex-specific, with female colonists being consistent while male colonists being flexible in their behaviour.

Recently, we reported similar sex-specific patterns of plasticity/consistency of stress regulation in the same colonizing population of gerbils: elevation of glucocorticoid levels in first colonists rapidly dampened with colony age in males but remained high across subsequent generations of females [65]. Consequently, in terms of stress regulation, female colonists manifest a reactive strategy associated with high activity of the hypothalamic–pituitary–adrenal axis, while in terms of behavioural patterns, they display a proactive style, in agreement with the two-tier model [92–94] and labile relationships between glucocorticoid and behavioural variation [95]. A meta-analysis across 21 species indicates that individual hormone levels only weakly correlate with proactive behavioural traits (aggression, boldness, exploration and activity) and explain on average only 2% of personality variation [96]. Moreover, the direction of the causal link between stress regulation and behavioural traits depends on a species’ life history. In slow species, bolder individuals possess lower stress reactivity, whereas in fast species, bolder individuals have higher stress reactivity [95]. The midday gerbil is a small fast-living species with a short lifespan, early maturation and intense reproduction [57,58]; these characteristics may explain why female colonists combine the potentiated hypothalamic–pituitary–adrenal axis activity as an attribute of the reactive coping style with a proactive bold behavioural strategy.

Links between dispersal propensity and behaviour have been found in females but not in males also in great tits [16], and females are on average more consistent in behavioural traits (except for mate preferences) than males in personality studies [97]. We do not exactly know whether the revealed behavioural patterns of female colonists are true personality traits: we took only a few repeated measurements and, thus, could not partition intra- and inter-individual variation to assess individual consistency in behavioural responses [98,99]. On the other hand, earlier, we have noticed that in midday gerbils, behavioural traits (including boldness/exploration syndrome)—as assessed in the same test procedure in the laboratory—are highly repeatable and consistent across time and contexts, indicating distinct personalities [54,62]. Combined with the strong consistency of boldness/exploration levels in female colonists across generations, this observation suggests that the colonizer syndrome may be a heritable personality trait of females.

Another important inference from our work is that observations of colonization from first steps and at short time intervals allow to record short-term behavioural dynamics after colony establishment and detect changes, which otherwise can be missed. If we had not observed the colonization process from the beginning when the first settlers arrived, then we could have overlooked the abrupt alteration of male behaviour only 1 year after the colonies’ foundation and made wrong inferences about the sex-specific behavioural dynamics post-colonization.

In conclusion, sex differences in colonizing strategies remain an underexplored field of colonization research, and such an investigation expands the understanding of the evolution of dispersal and life-history strategies in males and females as well as the mechanisms underlying species range expansions. We discerned sex-specific patterns of behavioural variation between residents of the source population and first colonists and short-term dynamics of behavioural traits at first stages of range expansion. Females showed spatial sorting across an expanding range, with bold explorative colonizer phenotypes being found at the edge and retained in subsequent generations of colonist females. Males proved to not be behaviourally sorted, and their behaviour shifted towards shyer and less explorative as environmental uncertainty decreased in the ageing colonies. The flexible reversible response of male colonists to the changing environmental uncertainty and the rigid proactive colonizer syndrome in females represent two alternative strategies, both ensuring colonization success but in a sex-specific way. Observations of colonization from the first steps and at short time intervals enable researchers to record behavioural changes (in a colonist population after colony establishment), which otherwise can be missed. The overlooked short-term effects hinder detection of the flexibility of behavioural responses to changes in environmental uncertainty before, during and after colony foundation, thus calling for more studies conducted in real time. They can provide new insights into the mechanisms of colonization.

## Data Availability

The datasets generated and analysed during the current study and code are available as electronic supplementary materials [101].
